# Effects of collagen peptides intake on skin ageing and platelet release in chronologically aged mice revealed by cytokine array analysis

**DOI:** 10.1111/jcmm.13317

**Published:** 2017-09-18

**Authors:** Hongdong Song, Ling Zhang, Yongkang Luo, Siqi Zhang, Bo Li

**Affiliations:** ^1^ Beijing Advanced Innovation Center for Food Nutrition and Human Health College of Food Science and Nutritional Engineering China Agricultural University Beijing China; ^2^ College of Food Science and Nutritional Engineering China Agricultural University Beijing China; ^3^ Beijing Higher Institution Engineering Research Center of Animal Product Beijing China

**Keywords:** collagen peptides, action mechanism, chronologically aged mice, cytokine array, anti‐skin ageing, platelet release

## Abstract

Action mechanisms underlying various biological activities of collagen peptides (CPs) remained to be elucidated. Cytokines may play an important role in mediating these health benefits of CPs. This study aimed to systemically examine the cytokines in skin and blood regulated by CPs intake. Thirteen‐month‐old female Kunming mice were administered with CPs for 2 months (0 or 400 mg/kg bodyweight/day). The cytokines in skin and plasma were analysed using a 53‐cytokine array and corresponding ELISA kits. In skin, CPs intake significantly down‐regulated placenta growth factor (PIGF‐2), insulin‐like growth factor (IGF)‐binding protein (IGFBP) ‐2 and IGFBP‐3, and up‐regulated platelet factor 4 (PF4), serpin E1 and transforming growth factor (TGF)‐β_1_. CPs treatment also increased the type I collagen mRNA and protein levels and improved the aged skin collagen fibres. In plasma, nine cytokines were significantly down‐regulated by CPs intake compared to the model group: fibroblast growth factor (FGF)‐2, heparin‐binding (HB) epidermal growth factor (EGF)‐like growth factor (HB‐EGF), hepatocyte growth factor (HGF), platelet‐derived growth factor (PDGF)‐AB/BB, vascular endothelial growth factor (VEGF), chemokine (C‐X‐C motif) ligand 1 (KC), matrix metalloproteinase (MMP)‐9, interleukin (IL)‐1α and IL‐10; 2 cytokines were significantly up‐regulated, including TGF‐β_1_ and serpin F1. Furthermore, CPs intake significantly decreased the level of platelet release indicators in the plasma and washed platelets, including PF4, granule membrane protein (GMP)‐140, β‐thromboglobulin and serotonin. These results provide a mechanism underlying anti‐skin ageing by CPs intake and highlight potential application of CPs as a healthcare supplement to combat cancer and cardiovascular disease by inhibiting platelet release.

## Introduction

The incidences of many diseases, such as vascular diseases, cancer, arthritis and osteoporosis, rise rapidly with age 1, 2. Therefore, interventions that delay ageing or age‐related diseases would greatly benefit health. To date, healthcare supplements that delay ageing or age‐related diseases have received great attention. CPs, a hydrolysis product of collagen, have been widely used in food, cosmetic and pharmaceutical industries. As a healthcare supplement, CPs has a variety of interesting health benefits, such as antiatherosclerotic activity 3, antitumor activity 4 and preventing osteoporosis 5. In particular, much attention has been paid to the anti‐skin ageing activity of CPs in recent years. Many studies, from cell and animal experiments to clinical trials, have demonstrated the beneficial effects of CPs on skin ageing 6, 7, 8. However, the mechanism underlying the health benefits of CPs has not been entirely elucidated.

Cytokines are a broad category of low molecular weight proteins that are involved in cell signalling. They play important roles in regulating cell growth, differentiation, cell survival and apoptosis, and the expression of extracellular matrix and adhesion molecules. One of the biologic characteristics of cytokines is their functional pleiotropy, that is one cytokine exhibits various biological activities on different target cells, and their redundancy, that is several different cytokines show similar and overlapping actions on a certain cell 9. Considering that CPs intake has various health benefits, we hypothesize that the biological activities of CPs were mediated, at least partially, through effects on the production of cytokines. Indeed, a previous study has reported that the stimulation of longitudinal bone growth by CPs is mediated by increased expression of insulin‐like growth factor 1 (IGF‐1) and bone morphogenetic protein 2 (BMP‐2) 10. The cytokines, including growth factors, inflammatory cytokines and chemokines, play an important role in skin ageing 11. However, it still remains unclear about which cytokines in skin are regulated by CPs intake. In addition, after digested and absorbed in gastrointestinal (GI) tract, CPs was transported first into the blood. The cytokines in blood have a critical role in many physiological and pathological processes. Whether CPs intake has a regulatory role for the cytokines in blood, thus exerting its biological activities needs further study. Therefore, determining the cytokines in skin and blood that are significantly regulated by CPs intake helps to elucidate the action mechanisms of CPs and explore its potential functional activity.

It is difficult to obtain a comprehensive expression profile of cytokines in skin and blood, because traditional techniques such as Western blotting, enzyme‐linked immunosorbent assays (ELISA) and reverse transcriptase polymerase chain reaction only allow the analysis of a limited repertoire of proteins/cytokines. This may miss novel, unexpected changes in the cytokine expression profile. Although gene arrays and complimentary DNA arrays provide useful data about gene expression, these results does not always reflect the expression levels of the proteins they correspond to. Because proteins are the biological effector molecules, the protein level is physiologically more relevant 12.

Protein array is a high‐throughput method used to track the interactions and activities of proteins, and to determine their function on a large scale 13. It is a powerful tool to rapidly and simultaneously screen a large number of potential candidate proteins and has been widely used to determine the target protein regulated by a given component for understanding its action mechanisms. To our knowledge, no comprehensive study about the effect of CPs intake on body cytokine profile has been performed to date. Therefore, the objective of this study was to determine the cytokines that are regulated by CPs intake both in skin and blood through the use of a 53‐cytokine array and attempt to elucidate the action mechanisms of CPs and explore its potential functional activity.

## Materials and methods

### Materials and chemicals

Silver carp skin was supplied by Hubei Zhongke Agriculture Co., Ltd. (Jingzhou, China). Alcalase, papain and trypsin were purchased from Novozymes (Beijing, China). Collagen type I from rat tail tendon, pepsin from porcine gastric mucosa and pancreatin from porcine were purchased from Sigma‐Aldrich (Shanghai, China). Foetal bovine serum (FBS), Dulbecco modified Eagle's minimal essential medium (DMEM), penicillin, streptomycin, non‐essential amino acids (NEAA) and Hank's buffered saline solution (HBSS) were purchased from Gibco (Life Technologies, Grand Island, NY, USA). The BCA protein assay kit was purchased from Beijing Solarbio Science and Technology Co., ltd. (Beijing, China). Mouse cytokine array kits were obtained from R&D systems (Minneapolis, MN, USA). Commercial kits used for determination of TGF‐β_1_, VEGF, PF4, GMP‐140, β‐thromboglobulin (β‐TG) and serotonin (5‐HT) were purchased from Jiancheng Institute of Biotechnology (Nanjing, China). The mouse antibodies against TGF‐β_1_, type I collagen and GAPDH were purchased from Abcam (Cambridge, UK). Other chemicals used were analytical grade or better.

### CPs preparation

Briefly, the thawed silver carp skin was firstly treated in 0.05 M NaOH and then in 0.2% H_2_S0_4_ (1:6, w/v) at room temperature for 60 min. After acid treatment, the swollen skins were soaked in distilled water at 45°C for 12 hrs to extract gelatin. The resultant gelatin was enzymatically hydrolyzed by a mixture of three enzymes (papain, trypsin and Alcalase) for 4.0 hrs. The enzymatic hydrolysate was then dialysed with 200 Da membranes to obtain CPs. The molecular weight distribution and amino acid composition of CPs has been described in previous report 7.

### Animals, diets and treatments

All procedures involving experimental animals were performed in accordance with protocols approved by the Committee for Animal Research of Peking University and conformed to the Guide for the Care and Use of Laboratory Animals (NIH publication No. 86‐23, revised 1996). The experiment was approved by the Animal Experimental Welfare & Ethical Inspection Committee, the Supervision, Inspection and Testing Center of Genetically Modified Organisms, Ministry of Agriculture (Beijing, China), and it was conducted in the Experimental Animal Center, Supervision and Testing Center for GMOs Food Safety, Ministry of Agriculture (SPF grade, Beijing, China). Eight‐week‐old (young mice, 28 ± 2 g, SPF grade) and 13‐month‐old (old mice, 45 ± 5 g, SPF grade) female Kunming mice were purchased from Sibeifu (Beijing, China) Laboratory Animal Science and Technology Co., Ltd. (Beijing, China). All mice were housed in cages (3–4 mice per cage) and were allowed free access to normal AIN‐93M purified diet and water. Animal room was maintained at a temperature of 23 ± 2°C, relative humidity of 40–70%, and artificially illuminated with a 12‐h light/dark cycle. Three days after arrival, 13‐month‐old mice were equally distributed, based on bodyweight, into two groups (*n* = 10/group), including the model group (M) and CPs treated group (CPs). Eight‐week‐old mice were set as young controls (Y). In addition to free access to normal AIN‐93M purified diet and water, the CPs group was intragastrically administrated with 0.2 ml of CPs with a dose of 400 mg/kg bodyweight; and the model group and young group were intragastrically administrated with 0.2 ml normal saline. The mice were intragastrically administrated with experimental diet for 2 months. After 2 months, mice were sacrificed and dorsal skin and blood samples were collected for further treatment.

### Skin protein extraction

The dorsal skin sample (about 50 mg) was washed with cold normal saline at 4°C and then cut into small pieces (about 1 mm^2^) and powdered in a liquid nitrogen bath. The resultant powder was lysed with 1.5 ml tissue lysis solution (50 mM Tris pH 7.5, 150 mM NaCl, 1% Triton X‐100) containing protease inhibitor cocktail (Amresco, Solon, OH, USA) and 1 mM PMSF. The mixture was then homogenized and incubated on ice for 30 min. Homogenate was centrifuged at 14,000 *g* for 10 min. at 4°C with a refrigerated centrifuge (TGL‐185, Pingfan Co., Ltd, Changsha, China) to collect the supernatants. Total protein concentration was determined using the BCA assay kit (Solarbio). Equal amounts (300 μg) of total protein per sample were loaded on cytokine array kit.

### Plasma preparation

Plasma was prepared according to a previous method 14 with a modification. Preparation procedures were designed to prevent activation of blood cells during preparation, such as performing preparation with the use of anticoagulant at room temperature. Blood was collected into EDTA tubes and processed within 30 min. of collection. After anticoagulant treatment, blood was centrifuged at 150 g for 15 min. at room temperature to obtain platelet‐rich plasma (PRP). PRP was further centrifuged at 750 *g* for 15 min. to collect supernatant. Finally, plasma was obtained by centrifugation at 1000 *g* for 10 min. at 4°C to remove cells and cellular fragments. Equal amounts (50 μl) of plasma per sample were loaded on cytokine array kit.

### Cytokine array kit analysis

**Figure 1:**
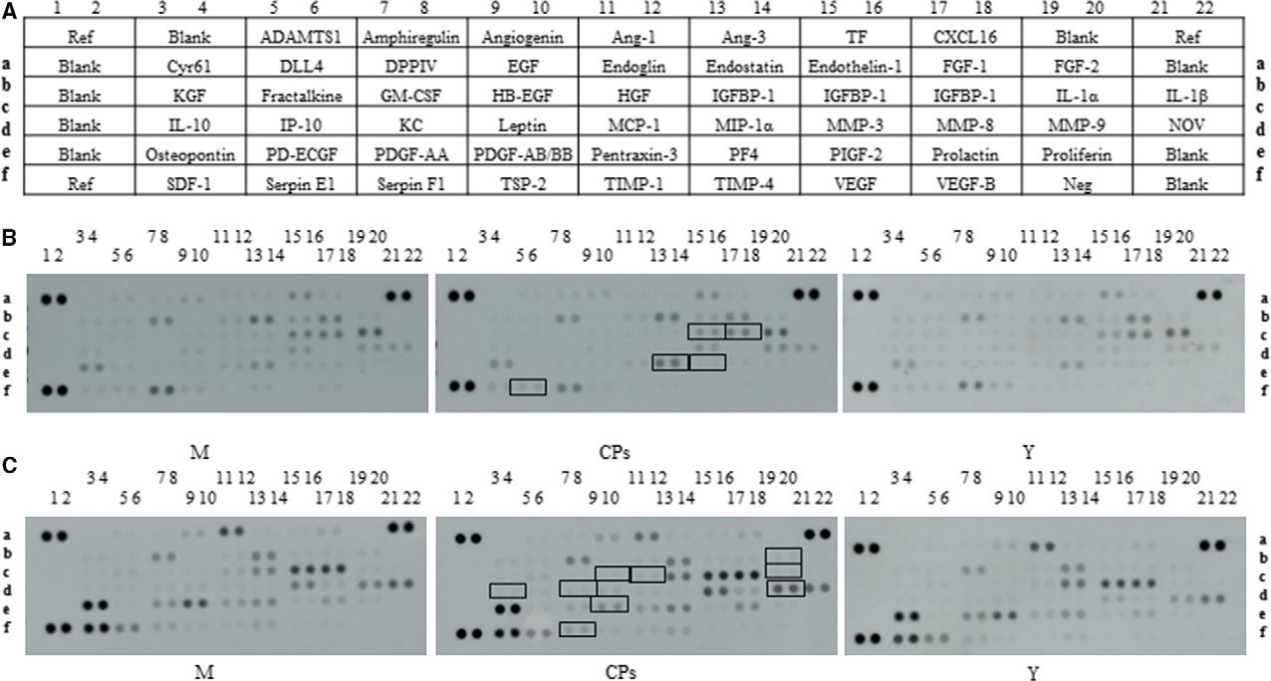
(**A**) The alignment of 53 cytokines on the Mouse Cytokine Array Kit. Ref, reference spots; Cyr61, cysteine‐rich angiogenic inducer 61; KGF, keratinocyte growth factor; IL, interleukin; SDF‐1, stromal cell‐derived factor 1; ADAMTS1, A disintegrin and metalloproteinase with thrombospondin motifs 1; DLL4, delta‐Like 4; IP‐10, interferon‐inducible protein‐10; PD‐ECGF, platelet‐derived endothelial cell growth factor; DPPIV, dipeptidyl peptidase‐4; GM‐CSF, granulocyte macrophage‐colony stimulating factor; KC, chemokine (C‐X‐C motif) ligand 1; PDGF, platelet‐derived growth factor; EGF, epidermal growth factor; HB‐EGF, heparin‐binding EGF‐like growth factor; TSP‐2, thrombospondin 2; Ang, angiopoietin; HGF, hepatocyte growth factor; MCP‐1, monocyte chemotactic protein 1; TIMP, tissue inhibitor of metalloproteinase; MIP‐1α, macrophage inflammatory proteins 1α; PF4, platelet factor 4; TF, tissue factor; IGFBP, insulin‐like growth factor‐binding protein; MMP, matrix metalloproteinase; PIGF‐2, placenta growth factor; VEGF, vascular endothelial growth factor; CXCL16, C‐X‐C motif chemokine ligand 16; FGF, fibroblast growth factor; Neg, negative control; NOV, nephroblastoma overexpressed. (**B**) Representative images of cytokine array blots probed with the skin samples; (**C**) Representative images of cytokine array blots probed with the plasma samples. Each blot represents immunoreactive staining against respective antibodies. Note the absence of staining at the negative control and blank slots. The relative expression levels of each cytokine were determined by comparing the pixel intensity of the respective blots to that of the positive control on the same array. The blots marked with black box are the cytokines that were significantly regulated compared to the model group. M, model group (old controls), CPs, administrated with collagen peptides, and Y, young controls.

The cytokine array kit (R&D systems) was used to simultaneously detect 53 cytokines (as shown in Fig. 1A) in each skin and plasma sample according to the manufacturer's instructions. Briefly, array membranes were incubated for 1 hr at room temperature in blocking buffer (all reagents were supplied with the array kit). After mixed with reconstituted detection antibody cocktails, samples were then incubated on the membranes overnight at 4°C on a rocking platform shaker. All of the following steps were performed at room temperature, and all wash procedures involved three washes in 1× wash buffer for 10 min. After incubated with detection antibody, membranes were washed and incubated with streptavidin‐conjugated horseradish peroxidase (1: 2000) for 30 min. on a rocking platform shaker. Unbound reagents were removed by washing, and the membranes were incubated in chemiluminescent detection reagents for 1 min. The chemiluminescent signal on each membrane was collected using an Amersham Imager 600 (GE Healthcare Life Sciences, Pittsburgh, PA, USA). The intensity (pixel density) of each spot on membrane was quantified using Image J software (National Institutes of Health, Bethesda, MD, USA), and corrected for background intensity and normalized to the membrane's positive control.

### ELISA analysis

Skin sample (about 30 mg) was cut into small pieces (about 1 mm^2^) and powdered in a liquid nitrogen bath. The resultant powder was suspended with 1.0 ml PBS containing protease inhibitor cocktail (Amresco). After thoroughly homogenized on ice, the mixture was centrifuged at 10,000 *g* for 10 min. at 4°C to collect the supernatants. Total protein concentration was determined using the BCA assay kit (Solarbio). TGF‐β_1_ was analysed using ELISA kit (Nanjing Jiancheng Bio Institute, Nanjing, China) according to the manufacturer's instructions, and the results were expressed in pg/mg protein.

The quantity of TGF‐β_1_, VEGF, platelet factor 4 (PF4), granule membrane protein‐140 (GMP‐140) and serotonin (5‐HT) in plasma was also determined by corresponding ELISA kits (Nanjing Jiancheng Bio Institute). The results were expressed in pg (or ng)/ml plasma.

### Western blotting

The skin protein extracts were transferred to PVDF membranes, blocked with 5% non‐fat milk in PBST and incubated with TGF‐β_1_ (Abcam), type I collagen (Abcam) and GAPDH (Abcam) antibodies at 4°C overnight. After washed by PBST, the resulting membranes were mixed with secondary antibodies and incubated for 1 hr at room temperature. Specific proteins were detected using an Amersham Imager 600 (GE Healthcare Life Sciences). Protein expression was quantified using ImageJ, and the results are expressed as density values normalized to the loading control (GAPDH).

### Real‐time PCR

Total RNA was extracted from mouse skin using tissue RNA kit (OMEGA, Norcross, GA, USA) following the manufacturer's protocol. The cDNA was synthesized from total RNA using M‐MLV First Strand cDNA Synthesis Kit (OMEGA) according to the manufacturer's protocol. Quantitative real‐time PCR was carried out on LightCycler 96 System (Roche, Basel, Switzerland) using a PCR kit (Amresco) according to the manufacturer's instructions. The primer sequences used in this study were listed as follows: collagen I, forward 5′‐GAGCGGAGAGTACTGGATCG‐3′, reverse 5′‐TACTCGAACGGGAATCCATC‐3′; GAPDH, forward 5′‐ TGCTGAGTATGTCGTGGAGTCTA‐3′, reverse 5′‐AGTGGGAGTTGCTGTTGAAATC‐3′ 15. The results were calculated to the GAPDH gene on the base of 2^−ΔΔCt^ algorithm.

### Histological analysis

The dorsal skins (approximately 1 cm^2^) were fixed in 4% neutral formalin, embedded in paraffin and sliced. Sections (thickness of 7 μm) were stained with haematoxylin–eosin (HE). The stained sections were analysed using an optical microscope.

### *In vitro* simulated gastrointestinal (GI) digestion and Caco‐2 cell absorption

After ingestion, CPs would be digested in the GI tract and absorbed into the blood through intestinal membrane. Therefore, in order to evaluate the effect of CPs on platelet granule release using *in vitro* model, a digested and absorbed CPs, named as CPsA, was obtained using *in vitro* simulated GI digestion and Caco‐2 monolayers. This method has been widely used and allows the prediction of oral compounds digestion and absorption in humans 16, 17.

For *in vitro* simulated GI digestion, CPs was digested enzymatically with pepsin and pancreatin according to a previously described method 18, with slight modifications. Briefly, CPs was dissolved in distilled water at a concentration of 4% (w/v) and incubated with pepsin (a ratio of enzyme to substrate of 1:50, w/w, and pH 2.0) at 37°C for 2 hrs. The pH was first adjusted to 5.3 with 0.9 M NaHCO_3_ and subsequently adjusted to pH 7.5 after adding 1 M NaOH. The solution was further hydrolysed with pancreatin (1:50, enzyme/CPs ratio, w/w) at 37°C for 4 hrs. Finally, the mixture was heated in boiling water for 10 min. to inactivate the enzymes. The resultant mixture were freeze‐dried and stored at −80°C until further treatment.

For the Caco‐2 cell absorption study, Caco‐2 cells (1.5 ml of 1 × 10^5^ cells/ml) were seeded in 6‐well filter support inserts (0.4 μm pore size, 23.1 mm diameter, 4.2 cm^2^ growth surface area, Nunc, Thermo Scientific, Waltham, MA, USA). Cell culture medium was replaced carefully every other day for 21 days until the Caco‐2 cells were differentiated fully as monolayers. Caco‐2 monolayers with a TEER higher than 800 Ω cm^2^ were used for absorption experiment 19. Briefly, after washed twice with pre‐warmed Hank's buffered saline solution (HBSS), the Caco‐2 cell monolayers were equilibrated for 30 min. at 37°C. Then, the apical HBSS was removed and an aliquot of 1.5 ml of CPs digest (15 mg/ml) from simulated GI digestion in HBSS was added on the apical side. The cell culture plates were then incubated at 37°C to start the absorption experiment. After 2 hrs, the absorbed permeates in the basolateral side were collected, lyophilized and desalinated. The desalinated product was named as CPsA and was used to evaluate its effect on platelet granule release.

### *In vitro* platelet release assay

Sprague–Dawley (SD) rats (280–320 g bodyweight, Vital River Laboratory Animal Technology Co., Ltd., Beijing, China) were intraperitoneally anesthetized with 2% sodium pentobarbital at a dose of 50 mg/kg of bodyweight. Blood was collected and mixed with sodium citrate to a final concentration 0.38%. The citrated blood was mixed with an equal volume of PBS, pH 7.4. PRP was obtained by centrifugation at 50× *g* for 10 min. at 23°C with a refrigerated centrifuge (TGL‐16A, Pingfan Co., Ltd). The PRP was again centrifuged at 50× *g* for 10 min. at 23°C. The resultant suspension was centrifuged at 750× *g* for 10 min. at 23°C to obtain the platelet pellet. The platelet pellet was washed once in HEPES/Tyrode's buffer (10 mM HEPES/NaOH, 5.56 mM glucose, 137 mM NaCl, 12 mM NaHCO_3_, 2.7 mM KCl, 0.36 mM NaH_2_PO_4_, 1 mM MgCl_2_, pH 7.4) in the presence of 1 mM EGTA 20. After centrifugation at 750× *g* for 10 min., the platelet pellet was gently suspended in HEPES/Tyrode's buffer and platelet concentrations were adjusted to 2–3 × 10^8^/ml.

The washed platelets were preincubated for 5 min. at 37°C. Platelet release was then induced for 10 min. by adding collagen at a final concentration of 50 μg/ml. 5 min. before adding collagen, CPsA was added to the washed platelet at a final concentration of 500 μg/ml. When reaction was finished, the samples were centrifuged at 10,000× *g* for 2 min. The supernatants were recovered and aliquoted. The levels of β‐TG and 5‐HT were determined using corresponding ELISA kits.

### Statistical analysis

All data were performed at least in triplicate. Results are expressed as means ± S.D.s. Comparisons between two groups were analysed by Student's *t‐*test. A difference was considered statistically significant when *P *<* *0.05.

## Results

### Cytokines in skin

**Figure 2:**
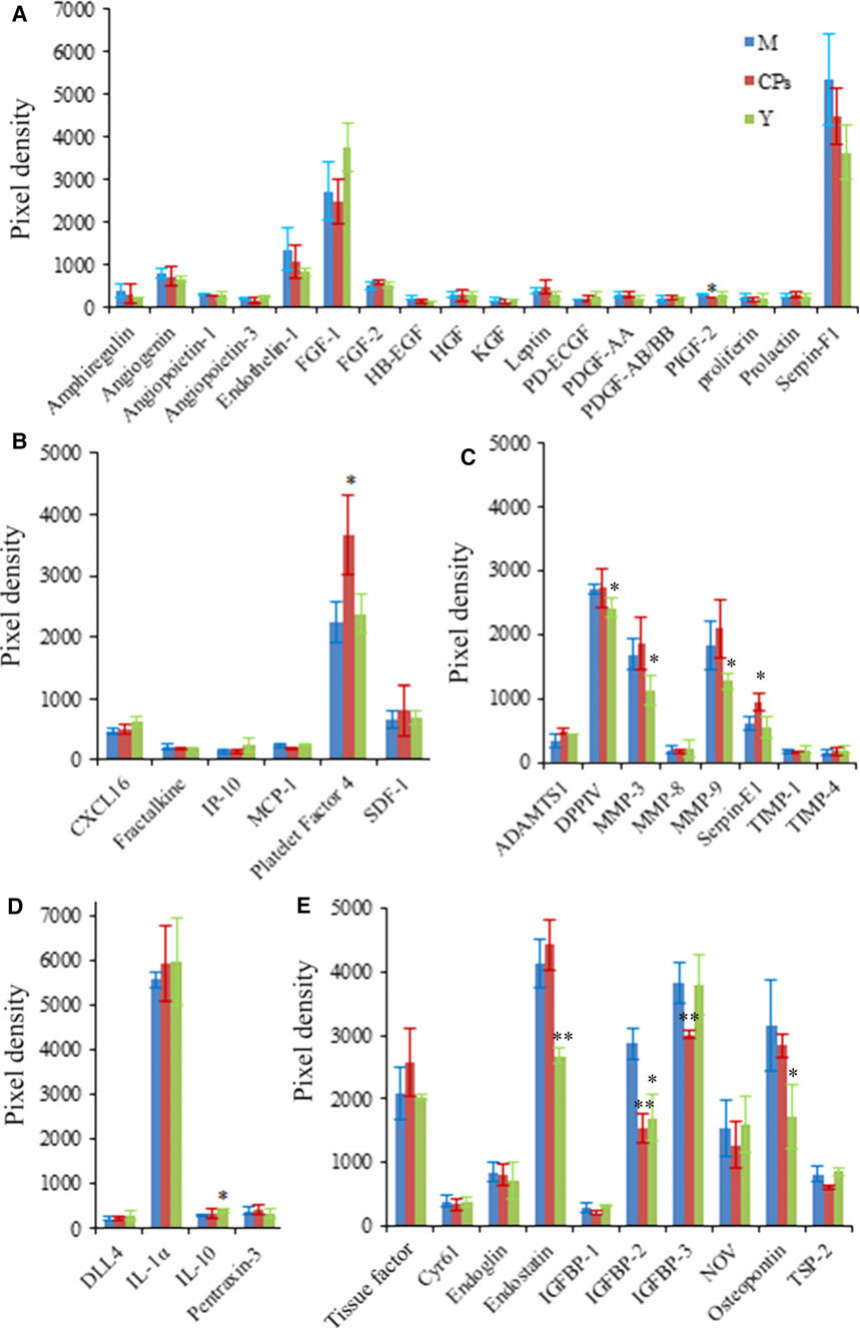
The effect of oral administration of collagen peptides (CPs) on cytokines in skin, (**A**) growth factors, (**B**) chemokines, (**C**) protease and inhibitors, (**D**) inflammatory cytokines, (**E**) other cytokines. M, model group (old controls), CPs, administrated with collagen peptides, and Y, young controls. Values are means ± S.D.s (*n* = 4 mice/group), * *P *<* *0.05, ** *P *<* *0.01, as compared with the model group.

**Figure 3:**
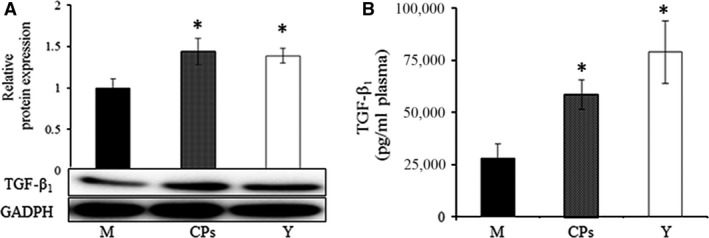
The effect of oral administration of collagen peptides (CPs) on TGF‐β_1_, (**A**) skin, (**B**) plasma. M, model group (old controls), CPs, administrated with collagen peptides, Y, young controls. Values are means ± S.D.s. (*n* = 3 mice/group), * *P *<* *0.05, as compared with the model group.

The level of certain cytokines in body organs and circulation system may change with age. Therefore, a young group was set to characterize the distinct cytokines profile between the model group and the CPs group. The alignment of the cytokine arrays is shown in Figure 1A. Examples of the array blots after incubation with the skin samples are shown in Figure 1B. Six reference spots (a1‐2, a21‐22 and f1‐2) exhibited the highest staining intensity, whereas the negative control (f19‐20) and all blank spots did not show above‐background staining. After corrected for background intensity and normalized to the membrane's positive control, 46 of 53 cytokines/proteins were detectable in skin by array analysis, whereas seven cytokines, including EGF, GM‐CSF, IL‐1β, KC, MIP‐1α, VEGF and VEGF‐B, were not detected by array analysis (not shown in Fig. 2). Based on their common functions, these cytokines/proteins are classified in five different groups: growth factors, chemokines, protease and inhibitors, inflammatory cytokines and other cytokines. Figure 2A shows the profile of detectable growth factors on the array. Only PIGF‐2 was significantly different between the model group and CPs group (*P *<* *0.05). PIGF‐2 level in the CPs group was reduced by 20% compared with that in the model group, indicating CPs intake significantly down‐regulated the level of PIGF‐2 in skin. In addition, considering the importance of TGF‐β in regulating skin collagen synthesis, TGF‐β_1_ was analysed by ELISA and Western blotting and the result is shown in Figure 3A (the ELISA result was consistent with that of Western blotting and was not shown). Compared to the model group, the TGF‐β_1_ level in the CPs group was significantly increased (*P *<* *0.05 *versus* the model group). For the chemokines on the array shown in Figure 2B, only platelet factor 4 (PF4) was significantly higher in CPs group than that in the model group (*P *<* *0.05). Figure 2C shows the profile of detectable protease and inhibitors on the array. Among them, serpin‐E1 had a significant difference between the CPs group and the model group (*P *<* *0.05). In addition, CPs intake also significantly down‐regulated IGFBP‐2 and IGFBP‐3 in skin (all *P *<* *0.01), but had no significant effect on four inflammatory cytokines, including DLL4, IL‐1α, IL‐10 and pentraxin‐3, as shown in Figure 2D and E. Several cytokine levels in the young group, including DPPIV (*P *<* *0.05), MMP‐3 (*P *<* *0.05) and ‐9 (*P *<* *0.05), endostatin (*P *<* *0.01), IGFBP‐2 (*P *<* *0.01) and osteopontin (*P *<* *0.05), were significantly lower than that in the model group, whereas IL‐10 in the young group was significantly higher than that in the model group (*P *<* *0.05).

**Figure 4:**
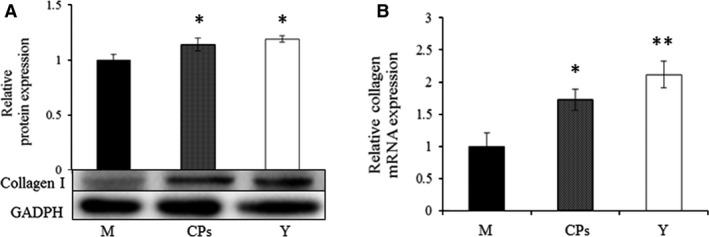
The Western blot analysis of type I collagen (**A**) and the evaluation of mRNA of collagen type I (**B**) in skin, M, model group (old controls), CPs, administrated with collagen peptides, Y, young controls. Values are means ± S.D.s. (*n* = 3 mice/group), * *P *<* *0.05, ** *P *<* *0.01, as compared with the model group.

The above results indicated that CPs intake had a beneficial effect on skin collagen synthesis. To test the speculation, the type I collagen in skin was analyzed by Western blot and the mRNA of type I collagen was evaluated by RT‐PCR. The results are shown in Figure 4A and B. As the positive control, the type I collagen content and mRNA level in the young group were significantly higher than that in the model group, which was consistent with previous reports 21, 22. The type I collagen content and mRNA level in the CPs group was 1.14‐fold and 1.72‐fold, respectively, as that of the model group, which demonstrated that CPs intake had a beneficial effect on the skin collagen synthesis.

**Figure 5:**
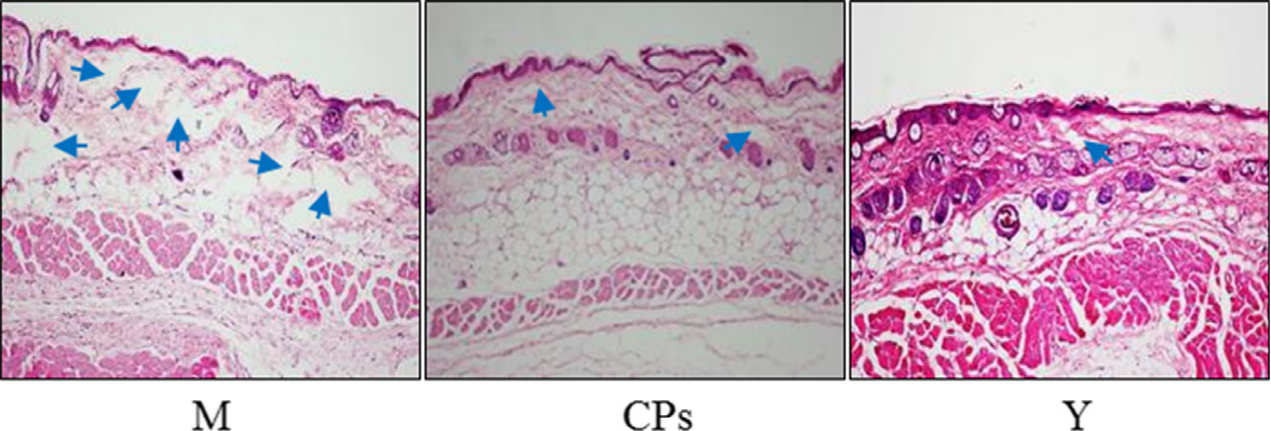
Images of mice dorsal skin section haematoxylin–eosin (HE) staining after CPs intake (200×). M, model group (old controls), CPs, administrated with collagen peptides; Y, young controls.

Further, skin biopsies with morphological examination were evaluated, and the images are shown in Figure 5. The skin collagen fibres were stained a light red with HE. In the model group (old controls), the lighter red and the more space (blue arrow) were observed in the dermis tissue than were those of the young group, which indicated that the aged skin appeared to be more sparse, fragmented and disorganized than did those of the young skin. After CPs intake, the space in the dermis tissue was decreased and the fibres appeared to be denser compared to the model group. These results demonstrated that CPs intake had a protective effect on the skin dermis fibres.

### Cytokines in plasma

**Figure 6:**
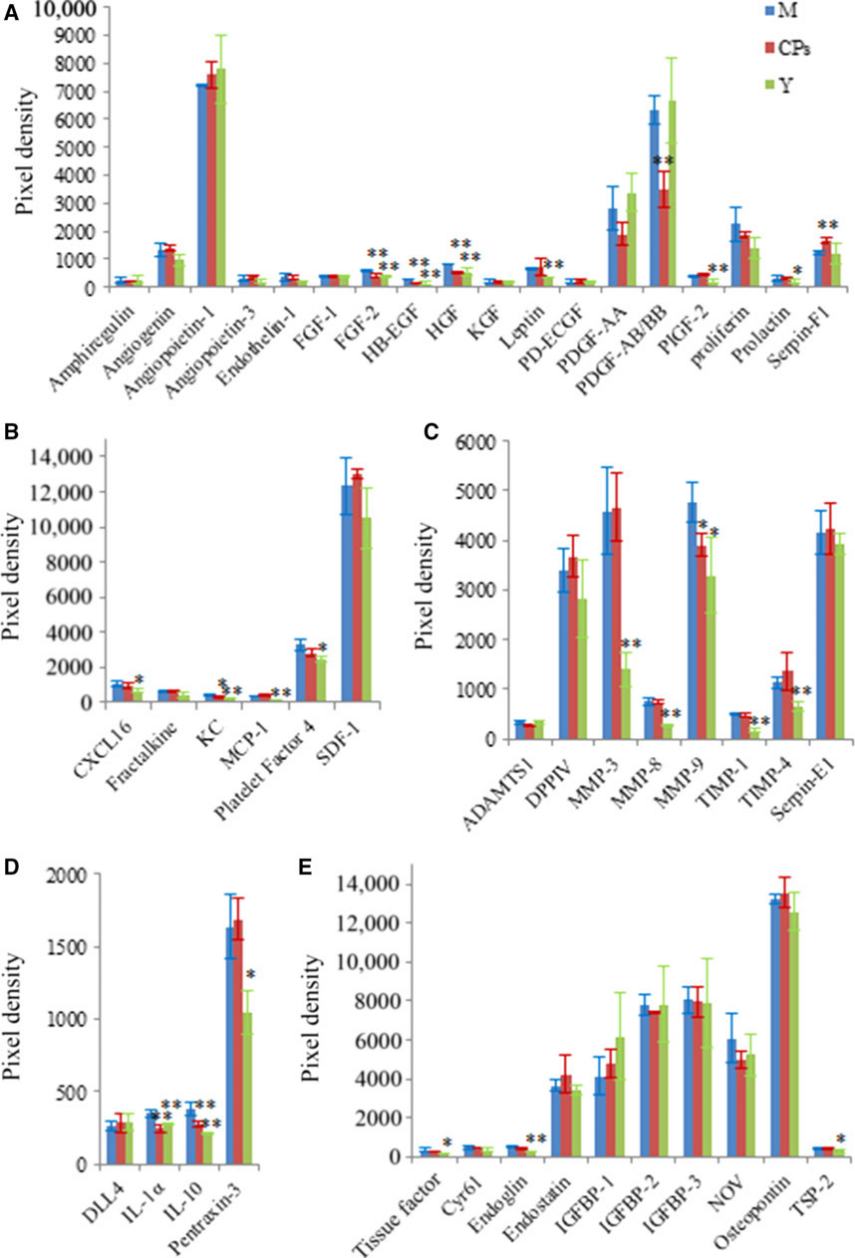
The effect of oral administration of collagen peptides (CPs) on cytokines in plasma, (**A**) growth factors, (**B**) chemokines, (**C**) protease and inhibitors, (**D**) inflammatory cytokines, (**E**) other cytokines. M, model group (old controls), CPs, administrated with collagen peptides, Y, young controls. Values are means ± S.D.s (*n* = 3 mice/group), * *P *<* *0.05, ** *P *<* *0.01, as compared with the model group.

**Figure 7:**
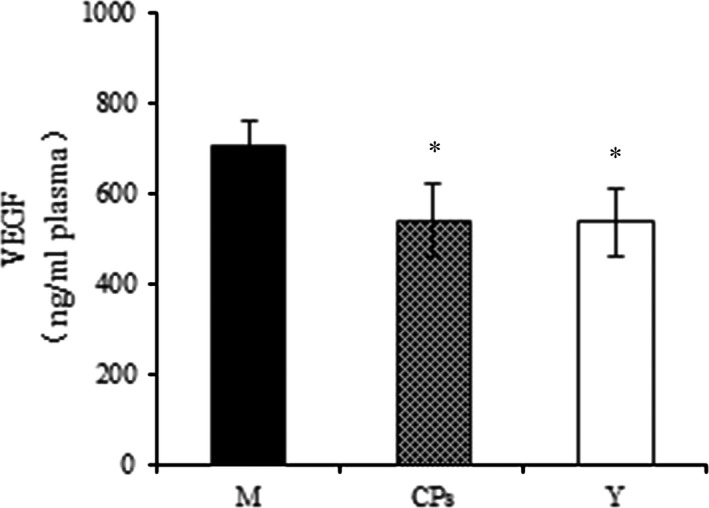
The effect of oral administration of collagen peptides (CPs) on VEGF in plasma. M, model group (old controls), CPs, administrated with collagen peptides; Y, young controls. Values are means ± S.D.s. (*n* = 3 mice/group), * *P *<* *0.05, as compared with model group.

Examples of the array blots after incubation with the plasma samples are shown in Figure. 1C. After corrected for background intensity and normalized to the membrane's positive control, EGF, GM‐CSF, IL‐1β, IP‐10, MIP‐1α, VEGF and VEGF‐B were not detectable by the present array analysis (not shown in Fig. 6). In the young group, 21 of 46 detectable cytokines on the array were significant lower than those in the model group (Fig. 6A–E), indicating that these cytokines were up‐regulated with age. However, eight cytokines were significantly down‐regulated by CPs intake compared to the model group: FGF‐2 (*P* < 0.01), HB‐EGF (*P* < 0.01), HGF (*P* < 0.01), PDGF‐AB/BB (*P* < 0.01), KC (*P* < 0.05), MMP‐9 (*P* < 0.05), IL‐1α (*P* < 0.01) and IL‐10 (*P* < 0.01). Serpin‐F1 was the only cytokine that was up‐regulated CPs treatment compared to the model (*P* < 0.01), as shown in Figure 6A. Considering the important role of TGF‐β_1_ and VEGF in collagen synthesis and angiogenesis, respectively, these two cytokines were analysed by corresponding ELISA kit. As shown in Figure 3B and Figure 7, the TGF‐β_1_ content in CPs group was significantly higher than the model group and reached that of young group (*P *<* *0.05 *versus* the model group and *P* > 0.05 *versus* the young group). However, CPs intake significantly down‐regulated VEGF in plasma compared to the model group (*P *<* *0.05).

### Platelet release indicators

**Figure 8:**
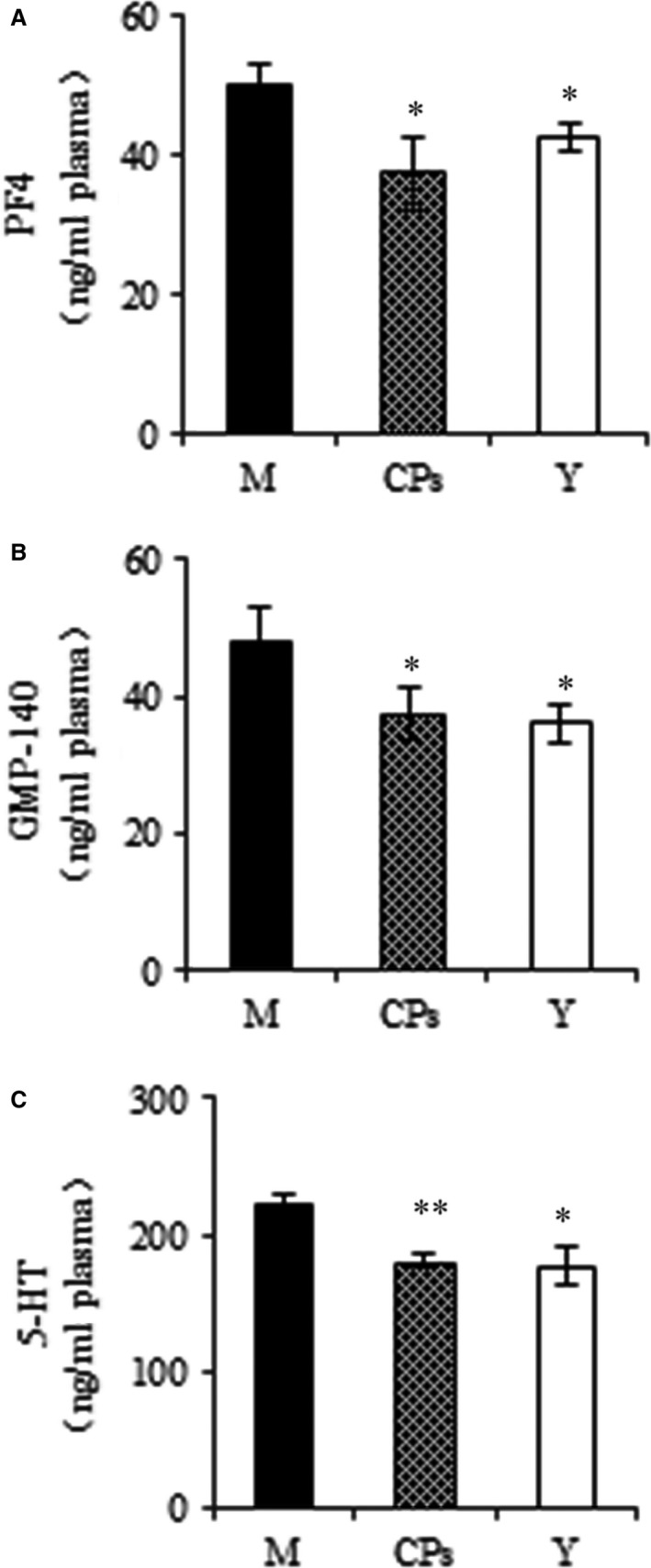
The effect of oral administration of collagen peptides (CPs) on platelet release indicators, (**A**) platelet factor 4 (PF4), (**B**) granule membrane protein 140 (GMP‐140), (**C**) serotonin (5‐HT). M, model group (old controls), CPs, administrated with collagen peptides; Y, young controls. Values are means ± S.D. (*n* = 3 mice/group), * *P *<* *0.05, ** *P *<* *0.01, as compared with the model group.

The markers of platelet release, PF4, GMP‐140 and serotonin (5‐HT) were analysed by corresponding ELISA kit. As shown in Figure 8A and B, the indicators of α‐granule secretion, PF4 and GMP‐140 were decreased after CPs was administered compared to the model group and reached the level of young group (*P *<* *0.05 *versus* the model group and *P *>* *0.05 *versus* the young group). This result indicated that CPs intake inhibited platelet α‐granule release. For the marker of dense granule release (Fig. 8C), 5‐HT was also down‐regulated after CPs treatment compared to the model group (*P *<* *0.01), which suggested that CPs intake inhibited platelet dense granule release.

**Figure 9:**
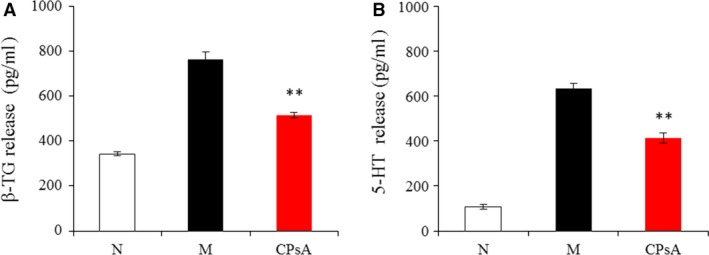
The effect of digested and absorbed collagen peptides (CPsA) on washed platelet release indicators, (**A**) β‐thromboglobulin (β‐TG), (**B**) serotonin (5‐HT). N, normal controls (treated by normal saline); M, model group (treated by 50 μg/ml collagen); CPsA, preincubated by digested and absorbed collagen peptides (CPsA, 500 μg/ml) and treated by 50 μg/ml collagen. Values are means ± S.D. (*n* = 3), ** *P *<* *0.01, as compared to the model group.

The effect of CPs on platelet granule release was further studied using *in vitro* assays, and the results are shown in Figure 9. β‐thromboglobulin (β‐TG) is another indicator of platelet α‐granule release. As shown in Figure 9A, as a potent platelet release activator, collagen significantly increased the washed platelet β‐TG release compared to the normal group. While CPsA pretreatment significantly decreased the released β‐TG level, which indicated that CPsA had an inhibitory activity on the platelet α‐granule release. Similarly, CPsA pretreatment also significantly decreased the released 5‐HT level compared to the model group (*P *<* *0.01). Overall, these data directly demonstrated that CPs exerted an inhibitory effect on platelet release reaction.

## Discussion

Ageing is the largest risk factor in the development of many diseases. The use of diet and oral supplements to prevent or decrease age‐related diseases has received increasing interest. Although the biological activities of CPs have been widely reported, few study was carried out in the chronologically aged model. In present study, 13‐month‐old Kunming mice, equivalent to 45 years old of human life 23, were employed to investigate the effect of CPs intake on cytokines in skin and blood. The CPs, used in the present study, has been demonstrated to have a stronger beneficial effect on the aging skin than other collagen hydrolysates (including gelatin and CPs with higher molecular weight) in our previous publication 7. The mice were administered with CPs for 2 months, equivalent to approximately 4.5 years of human life 23. After 2 months, we analysed 54 cytokines in skin and blood by cytokine array kit and ELISA kit and tried to elucidate the action mechanisms of CPs and explore its potential functional activity. To our knowledge, this study is the first report of comprehensive study about the effect of CPs intake on cytokine profile both in skin and blood.

### CPs and anti‐skin ageing

It has been well studied that skin ageing is closely related to cytokines, including growth factors, chemokines, inflammatory cytokines, MMPs and their inhibitors 11, 24. Therefore, we selected and analysed these related cytokines in skin by cytokine array kit. Six of 54 cytokines were significantly regulated after CPs intake compared to the model, including PIGF‐2, PF4, Serpin‐E1, IGFBP‐2, IGFBP‐3 and TGF‐β_1_. PIGF‐2 existed in skin in a low level and was significantly down‐regulated after CPs intake. As a member of the VEGF family, the physiological functions of PIGF‐2 were mainly focused on its proangiogenic activity 25. PF4 and serpin E1 are two cytokines that were significantly up‐regulated by CPs intake. There is very little knowledge about the association between these two cytokines and skin ageing. However, a previous study has reported that PF4 is an inhibitor of collagenase 26, and that serpin E1 controls the activities of MMPs, in particular MMP‐1, MMP‐2 and MMP‐3 by inhibiting tissue‐type plasminogen activator (t‐PA) and urokinase‐type plasminogen activator (u‐PA) 27. Three collagenases have been described in previous report, including collagenase‐1 (MMP‐1), collagenase‐2 (MMP‐8) and collagenase‐3 (MMP‐13) 28. These MMPs could degrade skin collagen, thus resulting in skin aging. The increased express level of PF4 and serpin E1 suggested that CPs intake could exert its beneficial effect on skin aging by inhibiting MMPs activity, although CPs intake had no significant effect on the expression level of MMP‐3, MMP‐8 and MMP‐9. It is noteworthy that MMP‐3 and MMP‐9 level in young group was lower than that in the model group, indicating that MMP‐3 and MMP‐9 expressions in skin were elevated with age. This result was consistent with previous reports 29, 30. Three insulin‐like growth factor binding proteins (IGFBPs) were also analysed on the array. Among them, IGFBP‐2 and IGFBP‐3 were significantly down‐regulated after CPs intake compared to the model group (*P *<* *0.01). IGFBP‐2 negatively regulated collagen synthesis by acting as an inhibitor of collagen gene expression by blocking IGF‐I action 31, 32. The level of IGFBP‐2 was significantly down‐regulated after the CPs was administered, indicating that CPs intake has a positive effect on skin collagen synthesis.

Reduction in collagen, the major structural component of skin, has been suggested as a cause of skin ageing 33. Alterations in skin collagen depend on the balance between collagen synthesis and degradation. Studies have demonstrated that natural ageing process decreases collagen synthesis and increases the expression of collagen‐degrading MMPs 34. Therefore, increasing collagen synthesis and/or decreasing MMPs were considered to be an effective protection against skin ageing. During the process of collagen synthesis, transforming growth factor‐β_1_ (TGF‐β_1_) is a critical regulator and stimulates collagen synthesis through the TGF‐β/Smad signalling system. Therefore, we analysed TGF‐β_1_ level in skin by ELISA kit and Western blot and found that TGF‐β_1_ was significantly up‐regulated after CPs intake. Studies have reported that ingestion of CPs led to an increase in skin collagen (especially of type I collagen) content in animal experiments and clinical trials with an unclear action mechanism 35, 36. The present result demonstrated that the TGF‐β_1_ level was significantly up‐regulated and reached that of the young mice after CPs intake. The up‐regulated TGF‐β_1_ may result in the increased skin collagen synthesis. Indeed, the type I collagen mRNA and protein levels were both increased by CPs treatment. It has been reported that collagen fibres make up 70–80% of the dermis, their primary location within the skin and the collagen production is decreased in chronologically aged skin 22, 37. CPs ingestion had a beneficial effect on the skin collagen production, which indicated that CPs intake had a protective effect against on the chronologically aged skin. The aged skin tissue appeared to be more sparse, fragmented and disorganized than did those of the young skin. Whereas the aged fibres in skin dermis were significantly improved after CPs intake, which demonstrated that CPs intake had an anti‐skin ageing activity.

### CPs and antiplatelet release

The cytokine profile regulated by CPs intake in the plasma was different from that in the skin. Eleven of 54 cytokines in the plasma were found to be regulated by CPs intake compared to the model group. Unexpectedly, FGF‐2, HB‐EGF, HGF PDGF‐AB/BB and VEGF were significantly down‐regulated by CPs ingestion instead of being up‐regulated. As important mitogens, these growth factors play crucial roles in tissue repair and angiogenesis. It has been widely reported that FGF‐2, HB‐EGF, HGF PDGF‐AB/BB and VEGF are important proangiogenic factors. Among them, VEGF and FGF‐2 are the most potent proangiogenic factors 38, 39, 40. In contrast, serpin F1 acts as antiangiogenic factor as reviewed in previous study 41. TGF‐β_1_ exhibits biphasic effect on angiogenesis: at relatively high concentrations (5–10 ng/ml), TGF‐β_1_ inhibited endothelial cell invasion and capillary lumen formation; at lower concentrations (100 pg/ml–1 ng/ml), TGF‐β_1_ potentiated the effect of FGF‐2‐ and VEGF‐induced invasion 42. The content of TGF‐β_1_ in plasma was significantly increased to 58,470 ± 7067 pg/ml plasma after CP treatment, which indicated that CPs intake had an antiangiogenic effect. KC, MMP‐9 and IL‐1α, known as proangiogenic factors, were also significantly down‐regulated by CPs intake. Therefore, based on the present results, CPs intake had an antiangiogenic activity through regulating the angiogenic factors in blood.

Although platelets are best known for their role in haemostasis, a substantial amount of data supports the idea that platelets play important roles in angiogenesis. Platelets are a reservoir for angiogenesis stimulators and inhibitors that are secreted in a differentially regulated process 43. Therefore, the present results directed us to consider whether CPs intake had a regulative effect on platelet, thus leading to changed level of these cytokines in plasma. PDGF has three isomorphic molecules: PDGF‐AA, PDGF‐AB and PDGF‐BB. PDGF‐AB and PDGF‐BB are stored mainly inside platelet α‐granules, whereas PDGF‐AA is produced, for example, by human osteosarcoma cells 44. In addition, many studies have reported that growth factors EGF, VEGF, IGF, FGF‐2, TGF‐β and HGF, chemokines KC, MCP‐1 and MIP‐1α, protease MMP‐9, and inflammatory cytokines IL‐1α/β, IL‐8 and IL‐10 are also released by platelet 45, 46, 47. Among them, eight cytokines, including PDGF‐AB/BB, FGF‐2, HGF, VEGF, KC, MMP‐9, IL‐1α and IL‐10, were significantly down‐regulated after CPs treatment. Therefore, we speculated that CPs intake had an inhibitory activity on platelet release, thus leading to decrease level of these cytokines in plasma.

In order to test the hypothesis, the markers of platelet release were analysed by corresponding ELISA kit. Platelets contain three major types of secretory organelles: α‐granules, dense granules and lysosomes. These three specific granule populations store different types of constituents. During circulation, platelets are reactive to various stimuli and release the materials stored in the specific granules. The α‐granules and dense granules have gained great attention because of their important role in haemostasis, tissue repair, angiogenesis and thrombosis. It has been widely adopted that PF4, β‐TG and GMP‐140 (P‐selectin or CD62P) are the markers of α‐granule secretion and serotonin (5‐HT) of dense granule 46. CPs intake significantly decreased the level of PF4, GMP‐140 and 5‐HT in plasma, indicating CPs had an inhibitory activity on platelet release. It is noteworthy that the level of PF4, GMP‐140 and 5‐HT in young group was significantly lower than that in the model group. This result demonstrated that platelet release was enhanced with age, which is consistent with previous report 48. Further, the antiplatelet release effects of CPs were directly demonstrated using *in vitro* assays. The released β‐TG and 5‐HT of washed platelet were significantly inhibited by CPsA pretreatment. Overall, we concluded that CPs had an inhibitory activity on platelet release.

Platelets are an extremely reactive cell of the circulatory system. In addition to exert haemostatic activity, platelets are also involved in the pathophysiology of several diseases, such as atherosclerosis, thrombosis and tumor, through the release of cytokines and some small molecules. Atherosclerosis and thrombosis are two common cardiovascular diseases. Platelets not only participate in the initiation and progression of atherosclerotic plaques, but also in thrombus complications of atheromastosus injury by α‐granules and dense granules release. The dense granules are rich in ADP, 5‐HT and Ca^2+^, which has a special importance in the amplification of the platelet activation 49. The activated platelet can join intact endothelial cells, which contribute to the development of atherosclerosis 50. On the other hand, the α‐granules contain great amounts of proteins, such as fibrinogen, FVW and PF4, which participate in platelet aggregation 49, thus contributing to thrombosis. The platelet α‐granules and dense granules release were significantly inhibited after CPs treatment. This result provided a novel perspective on preventing or treating atherosclerosis and thrombosis‐related cardiovascular diseases by CPs intake.

Angiogenesis is of key importance in the process of tumor progression. Platelet α‐granule is a rich source of many proangiogenic factors, including VEGF, PDGF, EGF and others 46. CPs intake had an inhibitory activity on the release of platelet α‐granule, which indicated that CPs may have antitumor activity. Indeed, a previous has reported that CPs intake extended the life span and inhibited spontaneous tumor incidence in Sprague–Dawley rats 4. The present study provides a mechanism to explain the antitumor activities of CPs.

In conclusion, the present study found the cytokines that were significantly regulated by CPs intake in skin and blood. These determined cytokines suggested a positive effect on skin collagen synthesis and an inhibitory activity on platelet release by CPs intake. These results provide a possible mechanism underlying anti‐skin ageing by CPs intake and highlight potential application of CPs as a healthcare supplement to combat cancer and cardiovascular disease by inhibiting platelet release.

## Conflict of interest

The authors confirm that there are no conflicts of interest.
